# Genetic structure and historical diversification of catfish *Brachyplatystoma platynemum* (Siluriformes: Pimelodidae) in the Amazon basin with implications for its conservation

**DOI:** 10.1002/ece3.1486

**Published:** 2015-04-22

**Authors:** Luz Eneida Ochoa, Luiz Henrique G Pereira, Guilherme Jose Costa-Silva, Fábio F Roxo, Jacqueline S Batista, Kyara Formiga, Fausto Foresti, Claudio Oliveira

**Affiliations:** 1 Departamento de Morfologia, Instituto de Biociências de Botucatu, Universidade Estadual PaulistaSão Paulo, Brazil; 2 Centro Interdisciplinar de Ciências da Vida, Universidade Federal da Integração Latino AmericanaFoz do Iguaçu, Brazil; 3 Laboratório Temático de Biologia Molecular, Instituto Nacional de Pesquisa da AmazôniaManaus, Brazil

**Keywords:** Biodiversity, climate oscillation, Madeira River, marine transgression, Neotropical fishes, phylogeography

## Abstract

*Brachyplatystoma platynemum* is a catfish species widely distributed in the Amazon basin. Despite being considered of little commercial interest, the decline in other fish populations has contributed to the increase in the catches of this species. The structure, population genetic variability, and evolutionary process that have driven the diversification of this species are presently unknown. Considering that, in order to better understand the genetic structure of this species, we analyzed individuals from seven locations of the Amazon basin using eight molecular markers: control region and cytochrome b mtDNA sequences, and a set of six nuclear microsatellite loci. The results show high levels of haplotype diversity and point to the occurrence of two structured populations (Amazon River and the Madeira River) with high values for *F*_ST_. Divergence time estimates based on mtDNA indicated that these populations diverged about 1.0 Mya (0.2–2.5 Mya 95% HPD) using cytochrome b and 1.4 Mya (0.2–2.7 Mya 95% HPD) using control region. During that time, the influence of climate changes and hydrological events such as sea level oscillations and drainage isolation as a result of geological processes in the Pleistocene may have contributed to the current structure of *B. platynemum* populations, as well as of differences in water chemistry in Madeira River. The strong genetic structure and the time of genetic divergence estimated for the groups may indicate the existence of strong structure populations of *B. platynemum* in the Amazon basin.

## Introduction

The Amazon and adjacent river basins is a phenomenal hot spot observed within Neotropical ichthyofauna and has been the focus of numerous studies (e.g., Goulding and Smith 1996; Araujo-Lima and Goulding 1997; Barthem and Goulding 1997; Barthem et al. 2003; Goulding et al. 2003a,b; Sistrom et al. 2009; Albert and Reis 2011). However, the ecological and geographic distribution of Neotropical fishes and their levels of biodiversity, especially intraspecific genetic richness, remain largely unknown (Beheregaray 2008).

Some tributaries of Amazon basin are known to have a distinct ichthyofauna composition, as well as the Madeira River one of the biggest tributaries of the Amazon River, draining about 850,000 km² of the South America platform. The white-water rivers draining from the Andes that are rich in sediments (e.g., Meta, Marañon, Napo, Madeira, etc.) have a distinct fish fauna compared with adjacent black-water rivers with low sediment charge draining from lowlands forested (e.g., Atabapo, Japurá, Tefé, Negro, etc.) (Albert and Reis 2011). Some hypotheses to explain these differences are related with water chemistry, as sediment charge, amount of oxygen, temperature, pH, and area of the annual flood (Henderson et al. 1998; Goulding et al. 2003a,b; Petry et al. 2003; Silva et al. 2003; Granado-Lorencio et al. 2005). These water characteristics can deeply affect biomass production (Lewis et al. 2001; Wantzen et al. 2002; Lindholm and Hessen 2007), electric conductivity, and visual ability with large implications in animal survival (Silva et al. 2003; Crampton and Albert 2006) and in species ranges among tributaries of the Amazon basin (Goulding et al. 1988).

Additionally, within the Amazon basin, studies using genetic data for understanding the history of fish populations (Hubert and Renno 2006; Hubert et al. 2006, 2007a,b; Renno et al. 2006) and the potential to discriminate between competing hypotheses of speciation (Sistrom et al. 2009) are really scarce. The underlying forces that have driven this huge diversification is a complex task, owing to this process cannot be restricted to a particular time interval or mechanism (Rull 2011). The diversification processes in South America have been tentatively explained by three main and nonexclusive hypotheses: the hydrogeological, paleontological, and climatic hypotheses (Hubert and Renno 2006).

**Figure 1:**
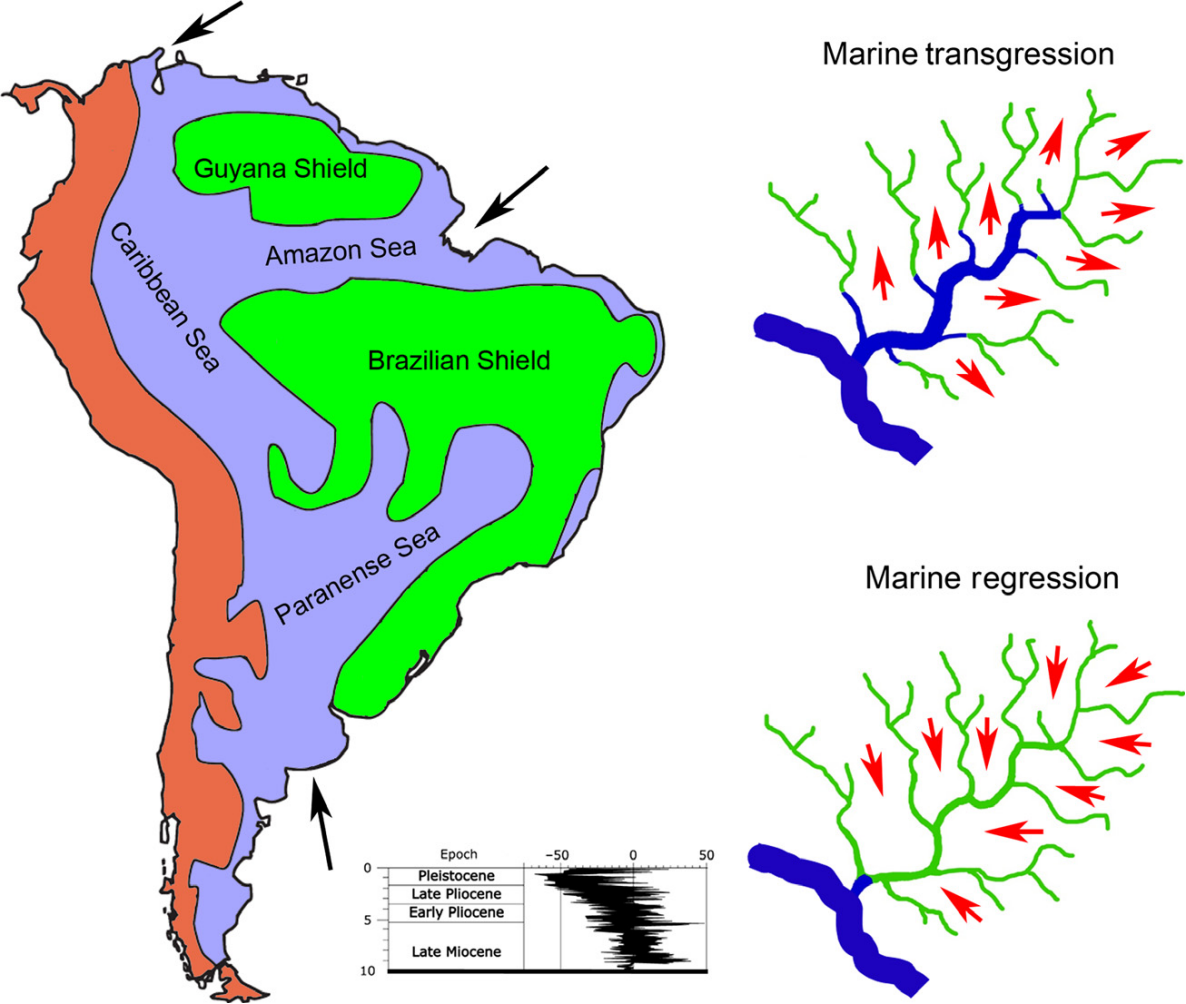
Hypothesis of marine transgression and regression in South America platform modified from Miller et al. (2005) and Ramos and Aleman (2000), and based on ideas of Hubert and Renno (2006) about marine transgression affecting the ichthyofauna composition of Amazon rivers. Black arrows indicated the three main lowland points in South America continent where sea can invade. Red arrows represent the fish migration to high lands in periods of marine transgression and migration to low land in marine regression.

Studies of phylogeography using molecular data within species provide a powerful tool for understanding the historical origins of the biodiversity, especially using paleogeography, paleobiology, and paleoclimatology data. Climatic change resulting in sea level variation (i.e., marine transgressions and regressions) also can deeply affect habitat taxonomical composition and species distribution (Roberts 1972). This impact could be more pronounced in South America than any other continent of the globe, considering to large areas of this continent have low elevation (<100 m) (Lundberg et al. 1998; Bloom and Lovejoy 2011). Throughout the Plio-Pleistocene, the climate oscillation was intensified resulting in sea level variations and marine invasions across the South America low lands (Hoorn 1993; Lovejoy et al. 1998; Lundberg 1998) with profound consequences to local fauna (Bloom and Lovejoy 2011). Marine incursions can isolate populations in uplands refuges accordingly to lead to diversification of lineages, followed by dispersal back to lowlands in marine water regression (Hubert and Renno 2006) (Fig.1). Whitemore and Prance (1987) suggested that within Pleistocene, in at least two times, the Madeira River formed a refuge area.

The climatic changes hypotheses are linked to the fluctuation of low and high sea level periods associated with glacial–interglacial periods, respectively. Low sea level events might be related with river interconnections at their lower section, enhancing freshwater taxa dispersal from one basin to another, while high sea level periods would have fragmented populations into newly isolated rivers basins promoting thus lineage diversification. Therefore, not only Pleistocene climatic changes but also earlier orogeny events (i.e., volcanic, tectonic, mountain uplift, alteration in drainage systems) occurring during the Miocene and Pliocene likely played an important role in the population divergence of South American freshwater fishes (Smith and Bermingham 2005; Swartz et al. 2007; Rull 2011; Roxo et al. 2012).

For aquatic organisms, phylogeography studies have indicated the role of geographic isolation across major South American drainages basins in structuring and generating biodiversity (Turchetto-Zolet et al. 2013). In the Amazon basin, the fishes represent not only a large biological diversity, but also have large economic implications for local populations, besides being an important source of protein for people living riverbanks.

Approximately 55% of the fish captured for the consumption are directed for to genus *Brachyplatystoma*, mainly species such as *B. rousseauxii*, *B. vaillantii*, and *B. filamentosum* (Barthem and Goulding 2007). The overexploitation of this group of species has generated the significant reduction of their populations, being currently rares in the artesanal fishing. Alternately other species have been included in the fishing for to support the consumption demand (Petrere et al. 2004), in this case, species such as *B. platynemum* have registered a increase in its capture since 2004 and in some countries such as Colombia, this species already has been recognized as endangered species (Mojica et al. 2002).

Therefore, the goal of our study was try to elucidate the genetic variation and the evolutionary process regarding to affect the evolution of *B. platynemum* in the Amazon River and in one of its main tributary (i.e., Madeira River), using sequences of the mtDNA and nuclear markers. We hypothesized that climate oscillation during Pleistocene resulting in marine transgression though low land of the South America continent and differences in water chemistry of the Madeira River, following to isolate local populations, and consequently leading genetic divergence.

## Materials and Methods

### Sample collection and DNA extraction

**Figure 2:**
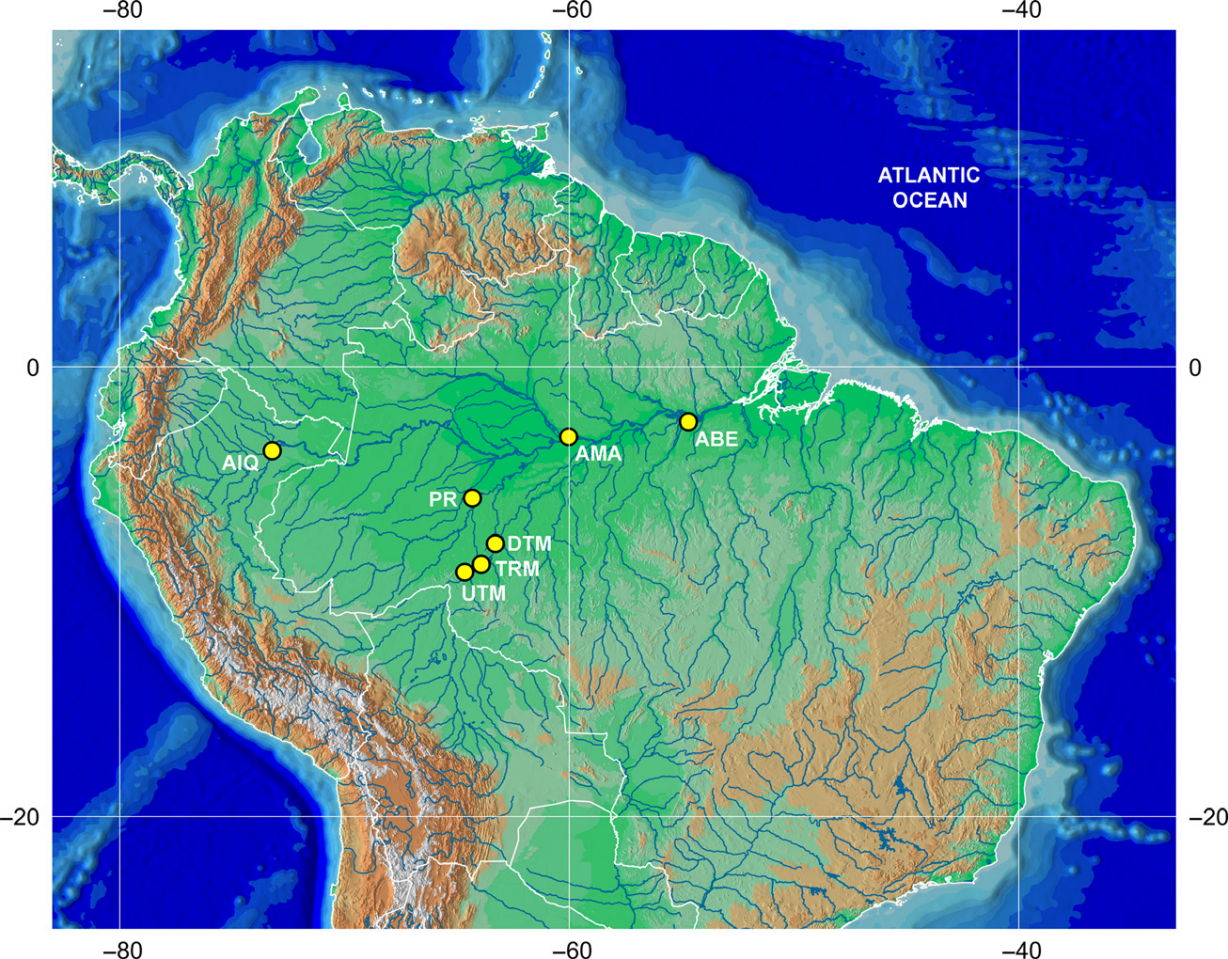
Sampling sites of *Brachyplatystoma platynemum;* AIP – Iquitos; PR – Purus River; UTM – Upstream Teotônio; TRM – Teotônio rapids; DTM – Downstream Teotônio; AMA – Manaus; ABE – Belém.

Muscle tissue and fin clips from *B. platynemum* individuals were collected in seven location of Amazon Basin, Iquitos (AIQ) (upstream Amazonas River), Purus River (PR), Manaus (AMA) (middle Amazonas River), Belem (ABE) (downstream Amazonas River), upstream Teotônio (UTM), Teotônio Rapids (TRM), and downstream Teotônio (DTM) and the last three in the Madeira River considered the most geographically complex tributary of the Amazon basin (Fig.2 and Table1). A total of 231 samples were obtained and were kept in 95% ethanol until extraction. Total DNA was extracted with a DNeasy Tissue kit (Qiagen) following the instructions of the manufacturer or following a modified NaCl extraction protocol adapted from Lopera-Barrero et al. (2008).

**Table 1:** Description of sample sites and number of samples analyzed for each molecular marker

Locations	Abbreviation	Basin		*N*	*N*_CR_	*N*_Cytb_	*N*_SSR_
Iquitos	AIP	Amazonas	3°43′39.65″S	30	27	27	30
			73°12′52.38″O				
Rio Purus	PR	Purus	5°50′22.03″S	38	31	30	38
			64°18′31.17″O				
Manaus	AMA	Amazonas	3°6′23.07″S	32	32	22	30
			60°1′35.15″O				
Upstream Teotônio	UTM	Madeira	9°7′41.85″S	22	22	8	20
			64°38′60.00″O				
Teotônio rapids	TRM	Madeira	8°46′60.00″S	40	35	24	33
			63°55′0.00″O				
Downstream Teotônio	DTM	Madeira	7°51′39.94″S	34	34	12	18
			63°17′16.42″S				
Belém	ABE	Amazonas	2°26′21.97″S	35	35	17	0
			54°41′55.45″O				

### Mitochondrial genes amplification and sequencing

Biogeographic analysis was performed using partial sequences of the mitochondrial control region (CR – 663 bp) for 216 samples and cytochrome b (Cytb – 1044 bp) for 140 samples. The amplifications of CR were performed with F-TTF primer by Sivasundar et al. (2001) and the reverse primer R3-3′TGGTAGCATGCGGGAAGAATGTCA5′ designed in this study. On the other hand, Cytb mitochondrial gene was amplified using the Cytb primers and conditions according to Pereira et al. (2011). Amplified products were checked on 1% agarose gels stained with Blue Green Loading Dye I (LGC Biotecnologia, São Paulo, Brazil). PCR products were purified using ExoSap-IT® (USB corporation, Cleveland, OH), sequenced using the “Big DyeTM Terminator v 3.1 Cycle Sequencing Ready Reaction Kit” (Life Technologies, São Paulo, Brazil), purified by ethanol precipitation, and loaded on an automatic sequencer 3130 Genetic Analyzer (Applied Biosystems, Life Technologies do Brasil Ltda, Sāo Paulo, Brazil) at Instituto de Biociências, Universidade Estadual Paulista, Botucatu, São Paulo, Brazil. DNA sequences were edited in the software BioEdit 7.0.5 (Hall^1999^) and aligned using the software Muscle (Edgar 2004).

### Microsatellite genotyping

Heterologous amplifications were performed using the microsatellite loci and PCR conditions developed by Batista (2010). PCR was performed in 10 *μ*L reaction mixture containing 2 *μ*L DNA template (10–50 ng/*μ*L), 1.0 *μ*L 10× PCR buffer (10 mmol/L tris-HCl, 50 mmol/L KCl, pH 8.4), 0.5 *μ*L forward *primer* (5 *μ*mol/L), 1.0 *μ*L *primer* R (5 *μ*mol/L), and 0.5 *μ*L *primer* M13 labeled at the 5′ with FAM, HEX, or NED fluorescent dyes, 2.1 *μ*L dNTPs (1 mmol/L), 0.30 *μ*L MgCl_2_ (50 mmol/L), and 0.21 *μ*L of Taq DNA polymerase 1U/*μ*L. The amplification of loci was performed using the following thermocycling conditions: initial denaturation at 92°C for 1 min, followed by 25 cycles at 94°C for 20 sec; 60–62°C for 40 sec; 68°C for 35 sec, followed by annealing of the primer using fluorescence 20 cycles at 93°C for 20 sec; and 53°C for 30 sec an extension to this cycle at 72°C for 35 sec. The final extension for the whole process of amplification was 72°C for 30 min.

The amplified products were checked on agarose gels (1.5%). According to the intensity of the bands of amplified products were made dilutions from 1:10 to 1:40. A total of 1 *μ*L of this dilution was used for genotyping with a solution containing 7.93 *μ*L Tween-20 (0.1%) and 0.07 *μ*L alleles size standard (TS 500) completing a final volume of 10 L.

Six loci were selected to show polymorphism (Table S1). The analyses were performed using two separate multiplex sets: M1: BR43, BR53, BR70 and M2: BR49, BR51, BR61. Amplicons were separated by electrophoresis on an ABI 3130xl DNA analyzer at LTBM/INPA (Laboratorio Tématico de Biólogia Molecular/Instituto Nacional de pesquisa da Amazonia), and their length was determined using GeneMapper software v.3.7 (Applied Biosystems) against an internal size standard (ET 500 GE Healthcare, São Paulo, Brazil).

### Analysis of genetic variability

The overall genetic variability based on mtDNA was estimated using the following parameters: number of haplotypes (H), private haplotypes (PH), haplotype diversity (Hd), number of polymorphic sites (S), total number of mutations (ETA), and nucleotide diversity (Pi) by DnaSP 5.1 software (Librado and Rozas 2009). To test recent population expansion or bottleneck in the population, we performed a selective neutrality Tajima's D test (Tajima 1989) and Fu's Fs (Fu 1997) with ARLEQUIN 3.11 (Excoffier et al. 2005).

The genetic variability among all six microsatellite loci were tested using the following parameters: number of alleles (A), expected and observed heterozygosity (*H*_E_, *H*_O_), deviation of Hardy–Weinberg equilibrium (HWE), inbreeding coefficient (*F*_IS_), *F* and *R* statistics, and allelic richness (Ar) obtained with POP-GEN 1.32 (Yeh et al. 2000) and ARLEQUIN 3.11. The software micro-checker 2.2.1 (van Oosterhout et al. 2004) was used to infer the most probable cause of HWE departures and the presence of null alleles (R).

### Analysis of genetic structure

The hierarchical analysis of molecular variance (AMOVA) and the fixation index (*F*_ST_) were conducted with 16,000 permutations to test the significance of pairwise population comparison implemented in ARLEQUIN 3.11 to both mitochondrial and microsatellites markers. The haplotype network was constructed for the mitochondrial markers in NETWORK 4.516 (Polzin and Daneshmand 2004). Bayesian clustering for the six microsatellites loci was also used to assess population relatedness and gene flow, using the program STRUCTURE 2.2 (Pritchard et al. 2000). The number of populations (K) was estimated using the admixture ancestral model with correlated alleles, allowing maximal population resolution, with K ranging from 1 to 8. Five independent runs of 500,000 Markov chain Monte Carlo (MCMC) generations and 100,000 generations of “burn-in” were used for each value of K. The true number of populations is expected to be the value of K that maximizes the estimated model log-likelihood, log (P(X|K)) (Falush et al. 2003), and the ad hoc statistic delta K (Evanno et al. 2005). Isolation by distance (IBD) was tested by computing the regression of FST/1-FST on geographic distances and the level of significance determined by performing a test with ISOLDE in GENEPOP 4.2 (Rousset 2008) based on 1000 randomization of the microsatellites data.

### Analysis of divergence times

The best fit partitioning schemes and the best nucleotide evolution model for each partition were evaluated in the software PartitionFinder (Lanfear et al. 2012) under the information-theoretic measure of Akaike information criterion (AICc).

Divergence dates were estimated by Bayesian coalescent analysis with BEAST 1.8.0 (Drummond and Rambaut 2007). For the analysis, we used the HKY+(I)+(G) model of nucleotide substitution as estimated in PartitionFinder (Lanfear et al. 2012). Three different coalescent priors as well as three molecular clock models were evaluated and tested using the Bayes factor (BF) as implemented in TRACER 1.5 (Rambaut and Drummond 2007a). A consensus tree was built using TreeAnnotator v1.8.0 (Rambaut and Drummond 2007b). Chains were run for 30 million generations each, sampling every 1000 iterations with the first 10% of trees discarded as burn-in. Taxonomic groupings were specified using the phylogenetic relationships of the *Brachyplatystoma* reported by Lundberg and Akama (2005). The Bleerker's genus group *Malacobagrus* is a subgenus within *Brachyplatystoma* that includes *B. filamentosum*, *B. capapretum*, *B. rousseauxii,* and *B. promagdalenae,* an extinct species dating to 11-12 ± 2.12 Mya (Lundberg 2005). We used this date as a minimum lower bound to date the split between the groups *Malacobagrus* and *B. platynemum*. The nature of the relationships between the genealogy of the sequences and the demographic history of the populations was characterized using the Bayesian skyline plot (BSP) model implemented in BEAST 1.5.4 (Drummond and Rambaut 2007; Drummond et al. 2005). For each BSP, the appropriate model of nucleotide substitution was determined using jModeltest (Darriba et al. 2012). Genealogies and model parameters for each lineage were sampled every 1000th iteration for 20 million generations under a relaxed molecular clock with uniformly distributed priors and a preburnin of 2000. Demographic plots for each analysis were visualized in Tracer v 1.5 (Rambaut and Drummond 2007a).

## Results

### Genetic diversity

#### Mitochondrial DNA

For the control region, 663 bp were sequenced from 216 individuals for the seven localities. A total of 66 haplotypes (H1-H66) were observed registering an overall haplotype diversity h - 0.931 ± 0.000. Considering all base positions, 618 were invariant, 45 were polymorphic sites, and 37 sites were parsimony informative. The total number of haplotypes between sites varied from H - 11 (UTM) to H - 18 (AIP) with absolute frequency of each haplotype between 1 and 21. Shared haplotypes were 19 (28.78%). Haplotypes 4, 5, and 8 were shared among more than one site, mainly of the Amazon River channel. The remainder was shared between two locations except the haplotypes 50, 52, 53, 55, 56, and 58, which were shared only among the Madeira River sites (DTM, TRM, and UTM). The highest haplotype diversity was recorded in AMA, h - 0.915 and AB h - 0.905. The Madeira River presented the lowest haplotype diversity (h - 0.635) compared to PR with a haplotype diversity of h - 0.892. All estimates of Tajima's D were positive except for TRM. However, none was significant (*P* > 0.05) (Table2).

**Table 2:** mtDNA control region (CR) and cytochrome B (Cyt b) genetic diversity of *Brachyplatystoma platynemum* estimated for each site

Sites	*N*		*H*		PH		*S*		*H*		*π*		Tajima'D	
	CR	Cyt b	CR	Cyt b	CR	Cyt b	CR	Cyt b	CR	Cyt b	CR	Cyt b	CR	Cytb
AIP	27	27	18	5	13	2	30	5	0.895	0.738	0.010	0.002	0.312	1.421
RP	31	30	14	3	8	1	24	7	0.893	0.545	0.008	0.002	1.313	0.812
UTM	22	8	12	6	4	3	26	12	0.900	0.893	0.008	0.004	0.114	−0.644
TRM	35	24	11	7	4	2	22	13	0.635	0.757	0.005	0.002	−1.446	−1.234
DTM	34	12	12	5	3	1	27	11	0.859	0.833	0.008	0.004	0.020	0.581
AMA	32	22	16	7	5	4	20	7	0.915	0.597	0.007	0.001	0.704	−0.711
ABE	35	17	17	4	10	1	21	6	0.906	0.625	0.007	0.001	0.625	−0.575

*N*, individuals; *H*, haplotypes; PH, private haplotypes; *S*, polymorphic sites; *h*, haplotype diversity; *π*, nucleotide diversity; Tajima'D, Tajima's D test.

**Figure 3:**
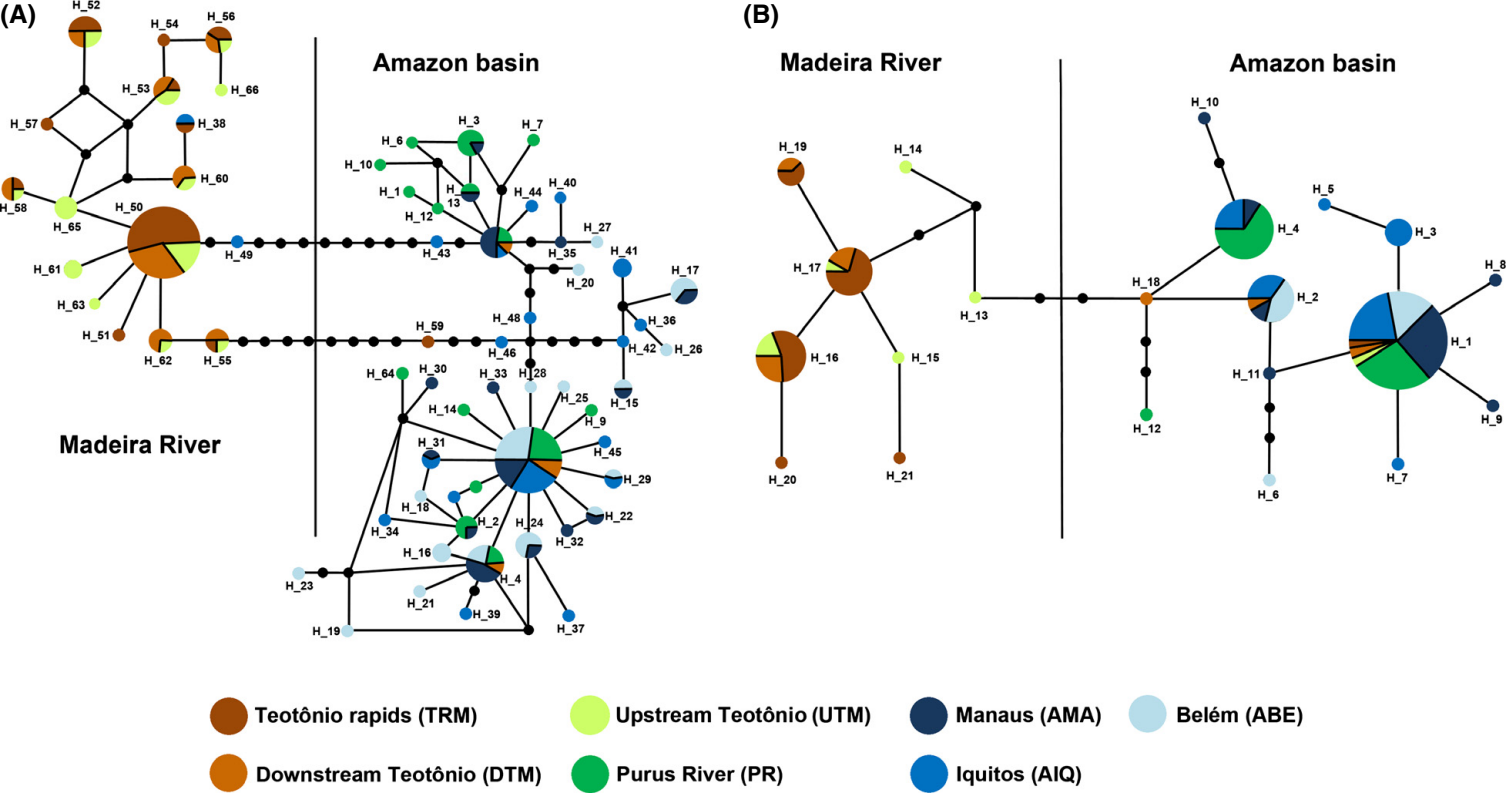
*Brachyplatystoma platynemum* statistical parsimony haplotype network for each mitochondrial marker (A) cytochrome b and (B) control region (CR). Circles size is proportional to the number of samples within a given haplotype. Circles are colored to reflect frequency of the haplotype in each designed geographic region (sampling sites). Black circles represent inferred mutational steps between haplotypes.

For the cytochrome *b* gene, a total of 140 individuals from seven sites were sequenced, and the total length of the alignment was 1044 base pairs (bp). Only 21 sites were polymorphic, defining a total of 14 unique parsimony informative haplotypes with absolute frequency of each haplotype between 1 and 16. The total number of haplotypes between locations varied from *H* - 3 to *H* - 7 for PR and AMA, respectively. Haplotype sharing was low among only six of the 21 haplotypes (28%) present in more than one site. The haplotype 1 was shared among all locations; haplotype 2, among AIQ, AMA, and ABE; haplotype 4, among AMA, PR, and AIQ; haplotype 16 and 17, among the three sites of the Madeira River (DTM, UTM, TRM); and haplotype 19, between DTM and TRM. Haplotype diversity ranged from *h* - 0.545 in PR to *h* - 0.893 in UTM, and in the same place of the highest haplotype diversity value (UTM) *h* - 0.893, the second largest number of private haplotypes (*H* - 3) was observed. Values of Tajima's D were negative, but none was significant (*P* > 0.05). Figure3 represents the haplotype network of two mitochondrial markers.

#### Microsatellites

The analysis comprised a total of 169 individuals and six loci from six sites. The micro-checker analyses indicated the occurrence of null alleles in three of the six loci from different locations analyzed (BR49, BR53, BR61). A total of 37 alleles were detected in all samples. Private alleles were recorded for the locations of AMA, AIQ, UTM, and TRM, which presented frequencies between 0.029 and 0.083. The number of alleles per locus ranged from 2 to 10 with an average of 6.43 alleles per locus (Table S2). The observed and expected heterozygosities ranged from *H*_O_ - 0.055 (BR51, DTM) to *H*_O_ - 0.864 (BR61, AIQ) and from *H*_E_ - 0.055 (BR51, DTM) to *H*_E_ - 0.849 (BR61, AIQ), respectively. Estimates of the inbreeding coefficient (*F*_IS_) ranged from 0 (BR51, UTM) to 0.867 (BR61, PR). The positive values for *F*_IS_ index suggested the existence of heterozygote deficiency in 18 of the 36 comparisons in the six locations analyzed, and these values could be derived from inbreeding or from the presence of a substructure within the population. Deviations from HWE were observed in 2 of the 36 estimates performed for the loci analyzed among populations after applying the Bonferroni correction (*P* < 0.007). The only loci with deviations were BR49 in AMA and BR53 in AIP. The significant values of HWE test were consistent with the estimates of the *F*_IS_ index and the deviations from *H*_O_ and *H*_E_ of each of the assessed locations indicating that low levels of heterozygosity were recorded (*H*_O_ < *H*_E_), and therefore, the values of the *F*_IS_ index were positive (Table S2). HWE departure is common with microsatellites (Alam and Islam 2005; Chevolot et al. 2006) and normally are associated with the occurrence of genotyping errors due to null alleles, stuttering or large allele dropout, and therefore we checked with the program micro-checker, and significant values due to stuttering or large allele dropout were not found. The estimates of occurrence of null alleles revealed positive values for two cases in which departure of HWE was identified. The occurrence of null alleles is a common problem in microsatellites studies and may be explained by the low efficiency of primer hybridization (Dakin and Avise 2004) and the possible differential amplification of alleles with different size (Wattier et al. 1998).

### Genetic structure

For the genetic structure initially analyzed, all sites were clustered as a unique group. The results of the AMOVA analysis showed a higher percentage of variation among the sites (53.35%) than within the sites (46.65%) with a *F*_ST_ - 0.533 (*P* < 0.05). The pairwise *F*_ST_ values of the two mitochondrial markers indicated substantial differentiation among the sites sampled in the main channel of the Amazon River and in the Purus River, compared to the sites of the Madeira River, with values ranging from 0.005 to 0.760 (*P* < 0.05) for the control region and from 0.007 to 0.779 for cytochrome b (Table3). Based on the *F*_ST_ results, the AMOVA test was conducted whit the samples conformed in two groups: the populations in the main Amazon River channel + Purus River (AMA, AIP, ABE and PR) and the Madeira River with its three sites (UTM, TRM, DTM). The result obtained revealed that most of the total molecular variance was partitioned between the two identified groups, supporting the *F*_ST_ values for pairwise comparisons. The results indicated that a low percentage of the total variance was attributed within sites (33.60% for control region and 29.21% for cytochrome b) (Table4) and showed an important percentage of variation among sites for the two markers (65.19% for CR and 69.48% for cytochrome b) (Table4). The statistical network (Fig.3) was conducted separately with each mitochondrial markers, and in both cases, the data confirmed the results of *F*_ST_ and AMOVA tests reveling two differentiated clusters in the sampling area for *B. platynemum*. One group identified like Amazon basin including the localities Iquitos (AIQ), Purus River (PR), Manaus (AMA), and Belem (ABE) and a second group including the three Madeira River localities (UTM, TRM, DTM).

**Table 3:** *F*_ST_ pairwise for *Brachyplatystoma platynemum* using control region (above) and cytochrome b (down). Significant values in bold (*P* < 0.002)

	AMA	AIQ	RP	UTM	TRM	DTM	ABE
AMA		0.027	0.025	**0.654**	**0.740**	**0.627**	0.005
AIQ	0.013		0.065	**0.568**	**0.672**	**0.545**	0.027
PR	0.114	0.046		**0.643**	**0.729**	**0.617**	0.142
UTM	**0.700**	**0.658**	**0.618**		0.009	−0.017	**0.682**
TRM	**0.779**	**0.751**	**0.721**	0.011		0.015	**0.760**
DTM	**0.653**	**0.618**	**0.580**	−0.074	0.008		**0.654**
ABE	0.018	0.027	0.147	**0.676**	**0.767**	**0.625**	

**Table 4:** Results of analysis of molecular variance (AMOVA). Control region (CR) and cytochrome b (Cytb) for *Brachyplatystoma platynemum* from Amazon and Madeira river groups

Source of variation	df *CR/Cytb*	Sum of squares *CR/Cytb*	Variance components *CR/Cytb*	Percentage of variation *CR/Cytb*
Among sites	1	514.66/162.13	4.84/2.65Va	65.19*/69.48*
Among sites within groups	5	26.134/10.42	0.09/0.05 Vb	1.20*/1.31*
Within sites	209/133	520.92/148.27	2.49/1.11 Vc	33.60/29.21
Total	215/139	1061.72/320.81	7.42/3.82	

* Significant values *P* < 0.05.

The genetic differentiation estimated with the six microsatellites loci among of the pairwise sites through the *F*_ST_ and *R*_ST_ indices displayed moderate values ranging from 0.001 to 0.239 and from 0.004 to 0.339, respectively. Some values were highly significant (*P* < 0.002, after Bonferroni correction), showing the existence of genetic differentiation among the samples analyzed (Table5). The AMOVA corroborated the values found for the *F*_ST_ and *R*_ST_ indices indicating that most of the total variance was attributed to variations within sites, but there was a significant percentage of variation among sites sampled (*F*_ST_ - 0.157, *P* < 0.01; *R*_ST_ - 0.093, *P* < 0.01) (Table6). The differences among indexes were probably due to differences in the mutation models on which they were based. While the *F*_ST_ is based on the IAM, the *R*_ST_ is based on the SMM. The *R*_ST_ index may be the best for microsatellites analyses, and it is expected that the values of *R*_ST_, under a SMM, would be higher than the values of *F*_ST_ (Slatkin 1995). However, as may be observed in our results, the *F*_ST_ values are usually greater than the *R*_ST_ values. This may be explained by the fact that probably not all microsatellite loci were evolving strictly under a SMM model (Balloux & Lugon-Moulin 2002). According to Slatkin (1995) and Balloux et al. (2000), departures of this pattern will result in a lower performance of *R*_ST_ in relation to *F*_ST_.

**Table 5:** *F* – statistics (above) and *R*-statistic (below) pairwise for *Brachyplatystoma platynemum* estimated with six microsatellites loci. Significant values in bold (*P* < 0.002)

	AMA	AIQ	PR	UTM	TRM	DTM
AMA	–	−0.010	**0.109**	**0.181**	**0.158**	**0.162**
AIQ	−0.039	–	**0.162**	**0.207**	**0.181**	**0.203**
PR	−0.124	−0.055	–	**0.290**	**0.253**	**0.246**
UTM	**0.164**	**0.205**	**0.197**	–	0.045	0.053
TRM	0.047	0.075	0.033	0.066	–	**0.073**
DTM	0.089	**0.145**	**0.141**	0.026	0.019	–

**Table 6:** Results of analysis of molecular variance (AMOVA) for *Brachyplatystoma platynemum* for six microsatellites loci analyzed

Source of variation	Sum of squares *F*_ST_*/R*_ST_	Variance components *F*_ST_*/R*_ST_	Percentage of variation *F*_ST_*/R*_ST_
Among sites	46.44/1233.42	0.32/8.29	15.75*/9.37
Among sites within groups	36.13/735.28	0.16/2.23	7.84*/2.52
Among individuals within sites	209.93/9289.55	0.19/6.40	9.64*/7.23
Within individuals	172.50/8552.00	1.35/71.52	66.76*/80.86
Total	464.99/19810.25	2.03/88.44	

*F*_ST_ - 0.157;

*R*_ST_ - 0.093;

* *P* < 0.01.

**Figure 4:**
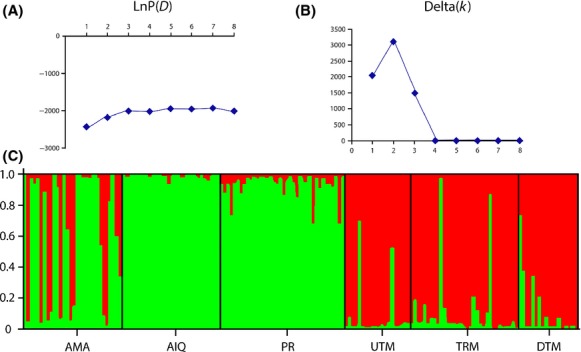
Bayesian clustering for the 6 microsatellite loci estimated in Structure 2.2 for *Brachyplatystoma platynemum*. (A) estimated LnP(D); (B) delta(k) showing the highest value in a population structure of *K* - 2; (C) Structure bar plot.

The structure analysis conducted under the admixture model and *K* - 1–8 populations showed the highest likelihood (ln(P)D) in a population structure of *K* - 2 (−2113.46 ± 0.171), a result corroborated by the estimation of ΔK (Fig.4).

The Mantel test using the microsatellites data did not show any relationship between the geographic distance and the values for the fixation index (*R*^2^ - 0.004) with *P* - 0.001, respectively, indicating that isolation by distance is unlikely to have generated the observed structure pattern.

### Divergence time

**Figure 5:**
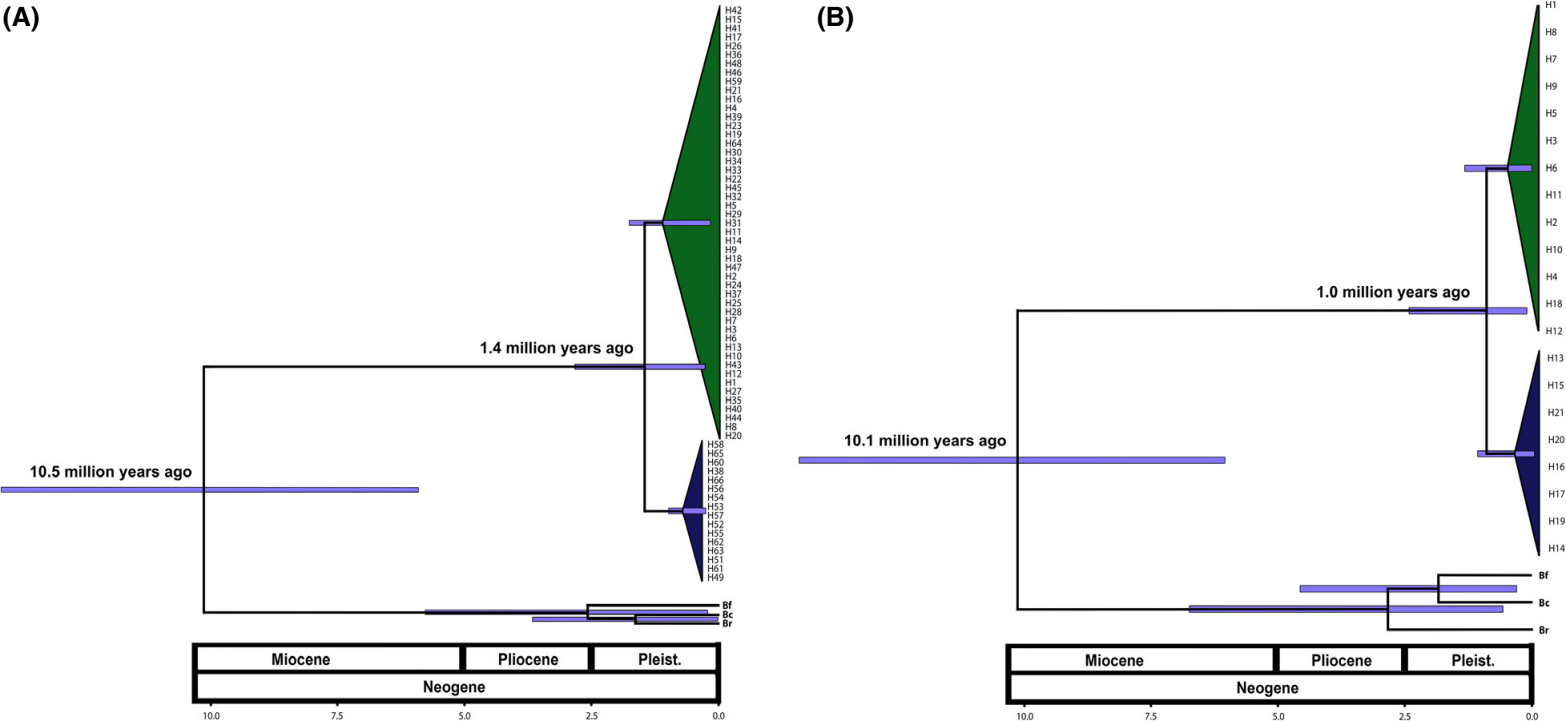
Divergence times of groups for *Brachyplatystoma platynemum* as estimated in BEAST. (A) Control region (Dloop). (B) Cytochrome b. Node bars display the 95% HPD. Time in the axis given millions of year before present. Amazon River group in green, Madeira River group in blue. Bf – *B. filamentosum*; Bc – *B. capapretum*; Br – *B. rousseauxii*.

**Figure 6:**
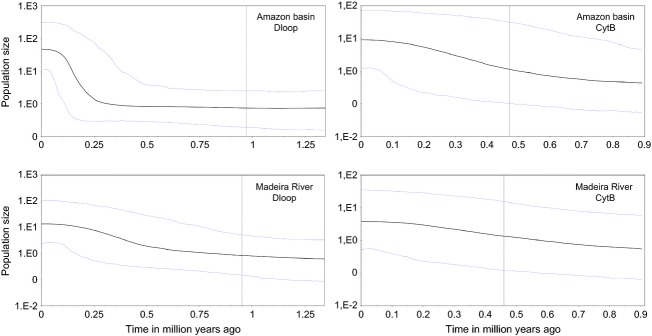
Bayesian skyline plot for each group of *Brachyplatystoma platynemum*. The black line represents the median value for the log of the population size (log Ne) and the blue lines represent the upper and lower 95% credible intervals. The *x*-axis measures time in substitutions per site per million years ago.

The difference in likelihood between the strict (control region - −2099.10; cytochrome *b* - −2454.27), relaxed lognormal (control region - −2098.90; cytochrome *b* - −2451.82), and the exponential (control region - −2091.64; cytochrome *b* - −2446.62) molecular clock showed that the exponential model was more adapted to our dataset for both markers. Similarly, the demographic tree prior test indicated that the constant size (BF - −2109.85) hypothesis cannot be rejected as compared with the exponential growth (BF - −2110.13) and expansion growth coalescent priors (−2110.00). Consequently, the relaxed exponential clock and constant population size prior were used to estimate the divergence time. The results show that the groups diverged during early Pleistocene 1.4 Mya (0.2–2.7 Mya 95% HPD) with control region (Fig.5A) to 1.0 Mya (0.2–2.5 Mya 95% HPD) with cytochrome b (Fig.5B). The values obtained for Fu's Fs, the more sensitive indicator of populations expansion, were negatives and highly significant for Amazon basin group (Fs_dloop_ - −31.341 and Fs_cytb_ - −2.333, *P* < 0.02) indicating an excess of low-frequency haplotypes, while in Madeira River group, these values were not significant (Fs_dloop_ - −3.522 and Fs_cytb_ - −1.202, *P* > 0.02). The demographic inference using the BSP model is summarized in Fig.6 which essentially plot N_e_T as a function of time; the uncertainty in the estimated parameters was evaluated using 95% highest probability density (HPD) intervals. The estimated expansion time of individual group was of 0.5–0.25 Myr (95%HPD) for Amazon basin and 0.5–0.45 Myr (95%HPD) for Madeira River. Significantly negative Fs values and Bayesian skyline plots depicting growth provide evidence that group Amazon basin have undergone recent expansion.

## Discussion

### Ecological hypothesis for genetic structure populations

The highest percentage of molecular variance was obtained for two groups of *B. platynemum* (i.e., indicating the existence of two structured populations), with the fixation index showing significant values between Amazon River and Madeira River. The *F*_ST_ values were supported by the statistical network; two weakly supported groups were identified with haplotype network, namely the Amazon River and the Madeira River (Fig.3); and it is possible to observe a low number of haplotypes share and the high number of mutations among groups. Despite recording lower levels of polymorphism at microsatellite loci, these analyses supported the pattern of structure found with the mitochondrial markers. The Bayesian analysis using Ln(P)D and delta de Evanno for the microsatellites corroborated the pattern observed, and the observed reduction of heterozygosity and the low deviation of Hardy–Weinberg equilibrium indicate that *B. platynemum* present a genetic structure probably like consequence of barriers to genetic flow followed for the genetic drift of their subpopulations.

This pattern is not an exception for species of this genus, and evidences of genetic structure had been observed in *B. filamentosum* associated with differences in water chemistry (Huergo 2009). However, for species as *B. rousseauxii* (Batista 2010), *B. vaillantii* (Formiga-Aquino 2004), and *B. capapretum* (Lira-Cordeiro 2013), structure populations in the Amazon basin had not been observed yet, probably as a result of the low number of genetic studies with Amazon catfishes (Hubert and Renno 2006; Hubert et al. 2006, 2007a,b; Renno et al. 2006).

The Madeira River is characterized with a large number of rapids and waterfalls (Torrente-Vilara et al. 2011), several times associated with population differentiation of some animal species (*Inia geoffrensis* Banguera-Hinestroza et al. 2002; *P. expansa,* Pearse et al. 2006) as well as some fish species (*Potamorrhaphis eigenmanni*, Lovejoy and Collette 2001). In this way, analysis of molecular variance (AMOVA) was realized independently among the localities sampling in the Madeira River (upstream (UTM), Teôtonio rapids (TRM), and downstream (DTM)); this analysis did not showed genetic structure, concluding that in this river exist a panmictic population. These results indicate that the Teotônio rapids (TRM) do not represent a physic barrier in gene flow for this species, as well as it has been identified for other fish species (e.g., *Brachyplatystoma* and *Pseudoplatystoma* in Goulding 1979; *Prochilodus nigricans* and *Colossoma macropomum* in Farias et al. 2010).

Furthermore, the Madeira River has a distinct water composition known as a white-water river draining from Andes (Albert and Reis 2011). Several authors (e.g., Henderson et al. 1998; Goulding et al. 2003a,b; Petry et al. 2003; Silva et al. 2003; Granado-Lorencio et al. 2005) have suggested that water chemistry could represent a barrier to gene flow limiting species ranges among Amazon basin (Goulding et al. 1988), whereas it can affect biomass production, electric conductivity, and visual ability with implications in animal survival (Lewis et al. 2001; Wantzen et al. 2002; Silva et al. 2003; Crampton and Albert 2006; Lindholm and Hessen 2007). This hypothesis can help to explain the different genetic structures of *B. platynemum* found between Amazon and Madeira rivers.

Patterns of genetic structure have been identified for other migratory species in the Amazonian basin as in *Prochilodus nigricans* involving locations of the Tapajós, Xingu, and Madeira rivers (Machado et al. 2008), *Semaprochilodus insignis* in Amazon basin and Orinoco (Passos et al. 2010), *Pseudoplatystoma corruscans* in the Paraná-Paraguay system (Pereira et al. 2009), and *B. rousseauxii* in Amazon basin (Batista and Alves-Gomes 2006). Within the last two species (i.e., *Pseudoplatystoma corruscans* and *B. rousseauxii*), the genetic structure was associated with the *homing* behavior, a process normally related with genetic segregation and association with geographic distribution. However, the Mantel test does not present evidences for isolation by distance among populations in our study. Additionally, we found a high genetic variability in the locality of Belem (ABE) (i.e., in the estuary region) and the localities of Iquitos (AIQ) and Purus River (APR). These results did not show pattern of segregation genetic, like is normally waited in the homing behavior.

Evolutionary biology and ecology assume that genetic diversity declines and differentiation increases toward the edge of a species' geographic range, where populations tend to be smaller and more isolated. Our results show a different pattern, and we find high genetic diversity between the localities more distant (Iquitos IQ and Belem BE) share most haplotypes mainly with the localities of Manaus and Purus rivers. In contrast, the localities sampled in the Madeira River recorded the lowest genetic diversity and the pairwise significantly higher genetic differentiation with other localities. Our results may indicate that the locality of Madeira River experiences higher population genetic drift and gene flow lower with other localities, possibly due to smaller population sizes, spatial isolation, and a more complex landscape structure.

### Sea level oscillation as a diversification process

The genetic structure identified in *B. platynemum* was supported by phylogenetic analyses based on the relationships of haplotypes of both mitochondrial markers. In agreement with the calibration of molecular clock, we found that the groups probably diverged about 1.4 Mya (0.2–2.7 Mya 95% HPD) using control region and 1.0 Mya (0.2–2.5 Mya 95% HPD) using cytochrome b (Fig.6). Patterns of diversification of populations and species in the Neotropical region have been mainly associated with the influence of marine incursions (Hubert and Renno 2006) and paleogeographic processes (Räsänen et al. 1990; Hoorn et al. 1995; Hubert et al. 2007b), mainly dating back to the Miocene. Significant and periodic sea level fluctuations have persisted throughout the last six million years, with an accelerated rhythm during the Pleistocene (Miller et al. 2005). The climatic changes associated with glacial–interglacial periods presented mainly two scenarios: during the low sea level periods might have allowed river interconnections at their lower section, enhancing freshwater taxa dispersal from one basin to another, while high sea level periods would have fragmented population into newly isolated river basins promoting the lineage diversification. In consequence during Pleistocene glaciations, the drainage isolation, habitat fragmentation, and poor dispersal ability have influenced the patterns of the population genetic structure of aquatic species. According to Farias and Hrbek (2008), more recent Pleistocene global sea level changes (Haq et al. 1987) could potentially have resulted in marine incursions or would have affected the extent of the Amazonian floodplain, especially in the eastern Amazon basin. Evidences of population genetic divergence within species occupying the Amazonian river basins were observed in other fishes species (*Potamorrhaphis guianensis*: Lovejoy and de Araujo 2000; *Serrasalmus rhombeus*: Hubert et al. 2007a; *Nannostomus unifasciatus*: Sistrom et al. 2009; *Paracheirodon axelrodi*: Cooke et al. 2009). In these studies, the population differentiation has been associated with ancient divergence events related with alteration of river drainage patterns and climatic changes during Miocene and Plio-Pleistocene. In this context, historical perturbation that in the past fragmented and/or altered the ecological structure of habitat and populations created new evolutionary pressures that either lead lineages to undergo extinction or to diversification.

### Madeira River as a refuge area during ice ages

The main factors responsible for climatic oscillation are CO_2_ concentration in the atmosphere, circulation of sea current, the positions of the continental, and the earth inclination (Budyko 1969; Petit et al. 1999; Rahmstorf 2003; Rothman et al. 2003). Whitemore and Prance (1987) suggested that within Pleistocene, in at least two times the Madeira River formed a refuge area mainly about 1.8 Mya to 11,000 years. This hypothesis predicts that climate changes limited the amount of available habitats for plants and animals during the ice ages due to the contraction of the rainforest (Haffer 1969; Prance 1982; Whitemore and Prance 1987) playing an important role in the search for the origin of high levels of local diversity and diversification of populations and in the maintenance of refuge areas minimizing the impact of stochastic extinction of species (Hubert et al. 2007a).

Recent studies indicated that the upper Amazon was colonized during the past 4 Mya, and only during the last million years, some of the tributaries were colonized (Aleixo 2004; Hubert et al. 2007b), while the current topology of the Madeira River was established during the past 2 Mya (Hubert et al. 2007a). Based on the theory of refuges and the data obtained for the divergence of *B. platynemum* groups (1.4 to 1.0 Mya), and also on the pattern of strong genetic structure observed, it is likely that climate changes isolated a population of this species in the Madeira River. According to Hubert et al. (2007a), *Serrasalmus rhombeus* show similar patterns of genetic structure with population divergence associated with climatic events in the Pleistocene (1 Mya) in the upper Madeira River, probably proceed from the Aripuanã refuge. Similar patterns were observed in other fish species, such as *Cichla* (Renno et al. 2006) and *Leporinus,* in French Guiana (Renno et al. 1990, 1991). The demographic analysis show that the genetic structure of *B. platynemum* contains signatures of demographic expansion consistent with Pleistocene glacial retreat. Specifically, the demographic analysis and genetic variation for the Amazon basin group show strong support for recent populations expansion represented by significantly negative Fs values and BSPs consistent with estimated times of these expansion range from 0.5 to 0.25 Myr (95% HPD). In contrast, the inferred demographic histories and divergence times of Madeira River group suggest that these populations were able to survive in refuge within colder regions, followed by demographic increases but without evidence of significant range expansion. It is probable that some ecological and chemistry barriers, perhaps between Amazon basin and Madeira River, have maintained the isolations of these populations. Processes of phenotypic differentiation between Amazon and Madeira River populations should not be discarded, but it requires to be confirmed by morphological analysis. However, owing to the small sample sizes for Madeira River group, these results require cautious interpretation, and we suggest that further sampling is needed in these localities before definit-ive statements about the demographic histories of these lineages can be made.

## Conclusion

In addition to other types of molecular studies, the limitations of the techniques (i.e., the use of microsatellites and mitochondrial DNA sequences), the large geographic ranges of some species, the stochastic effects of sampling, the difficult regarding to understanding species boundaries, and a poor taxonomy of many Neotropical fishes groups conspire to make phylogeographic studies with Amazonian fishes large challenging (Albert and Crampton 2003; Albert et al. 2004). As a consequence, a few number of population genetics studies have been applied to Neotropical freshwater fish species. However, our findings have important implications for the management and conservation prioritization of *B. platynemum*. We identified two distinct groups within this fish species, corresponding to two geographic regions (i.e., one for Amazon and Purus Rivers and other for Madeira River), probably resulted of differences of water chemistry within Amazon basin tributaries and of climate oscillation promoting marine transgression and regression during Pleistocene. Therefore, preservation of genetic diversity and evolutionary potential within this species will rely on ensuring that populations within each of these two lineages are targeted for conservation and be managed independently in fisheries statistics.

## Supporting Information

**Table S1.** Multiplex sets used for microsatellites genotype.

**Table S2.** Summary of microsatellite data for each site sampled of *B. platynemum*. *N*, number of individuals; *A*, allelic count; *H*_O_, observed heterozygosities; *H*_E_, expected heterozygosities; *F*_IS_, inbreeding coefficient; HWE, Hardy-Weinberg equilibrium; R, null allele.
