# Supplementary material for: Genetic structure and historical diversification of catfish Brachyplatystoma platynemum (Siluriformes: Pimelodidae) in the Amazon basin with implications for its conservation
Source: Ecol Evol. 2015 Apr 22;5(10):2005–20. doi: 10.1002/ece3.1486 (PMC4449755; doi:10.1002/ece3.1486)
Supplement: Supplementary file 1 [file ece30005-2005-sd1.doc]

| **Multiplex** | **Loci** | **size (pb)** | **Fluorescence** |
| --- | --- | --- | --- |
| **Multiplex 1** | BR43 | 143-161 | FAM |
|  | BR53 | 159-169 | HEX |
|  | BR70 | 207-215 | FAM |
|  |  |  |  |
| **Multiplex 2** | BR49 | 306-321 | HEX |
|  | BR51 | 296-304 | NED |
|  | BR61 | 261-271 | FAM |
