# Supplementary material for: Genetic structure and historical diversification of catfish Brachyplatystoma platynemum (Siluriformes: Pimelodidae) in the Amazon basin with implications for its conservation
Source: Ecol Evol. 2015 Apr 22;5(10):2005–20. doi: 10.1002/ece3.1486 (PMC4449755; doi:10.1002/ece3.1486)
Supplement: Supplementary file 2 [file ece30005-2005-sd2.docx]

|  |  |  | **Loci** |  |  |  |  |
| --- | --- | --- | --- | --- | --- | --- | --- |
| **Sites** | **Parameter** | **BR43** | **BR49** | **BR51** | **BR53** | **BR61** | **BR70** |
| **AMA** | ***N*** | 21 | 21 | 27 | 23 | 24 | 20 |
|  | ***A*** | 3 | 6 | 2 | 5 | 9 | 6 |
|  | **H_O_** | 0.714 | 0.476 | 0.370 | 0.609 | 0.750 | 0.750 |
|  | **H_E_** | 0.521 | 0.669 | 0.307 | 0.613 | 0.844 | 0.717 |
|  | ***F_IS_*** | -0.382 | 0.293 | -0.209 | 0.008 | 0.113 | -0.048 |
|  | **HWE** | 0.110 | **0.005** | 0.548 | 0.014 | 0.035 | 0.552 |
|  | ***R*** | - | 0.107 | - | - | - | - |
| **AIP** | ***N*** | 17 | 24 | 28 | 27 | 22 | 26 |
|  | ***A*** | 3 | 7 | 2 | 4 | 7 | 6 |
|  | **H_O_** | 0.470 | 0.542 | 0.286 | 0.296 | 0.864 | 0.654 |
|  | **H_E_** | 0.562 | 0.705 | 0.382 | 0.491 | 0.849 | 0.644 |
|  | ***F_IS_*** | 0.166 | 0.235 | 0.255 | 0.401 | -0.018 | -0.015 |
|  | **HWE** | 0.050 | 0.059 | 0.302 | **0.001** | 0.184 | 0.703 |
|  | ***R*** | - | - | - | 0.1254 | - | - |
| **RP** | ***N*** | 32 | 25 | 32 | 36 | 10 | 38 |
|  | ***A*** | 2 | 4 | 2 | 3 | 6 | 5 |
|  | **H_O_** | 0.469 | 0.440 | 0.469 | 0.250 | 0.100 | 0.710 |
|  | **H_E_** | 0.364 | 0.520 | 0.468 | 0.269 | 0.721 | 0.759 |
|  | ***F_IS_*** | -0.292 | 0.156 | -0.002 | 0.073 | 0.867 | 0.065 |
|  | **HWE** | 0.153 | 0.425 | 1.000 | 0.594 | 0.000 | 0.454 |
|  | ***R*** | - | - | - | - | 0.347 | - |
| **UTM** | ***N*** | 16 | 12 | 18 | 17 | 12 | 17 |
|  | ***A*** | 2 | 2 | 2 | 6 | 3 | 6 |
|  | **H_O_** | 0.500 | 0.167 | 0.055 | 0.529 | 0.583 | 0.529 |
|  | **H_E_** | 0.387 | 0.159 | 0.055 | 0.647 | 0.518 | 0.665 |
|  | ***F_IS_*** | -0.304 | -0.048 | 0.000 | 0.186 | -0.132 | 0.209 |
|  | **HWE** | 0.512 | 1.00 | 1.00 | 0.255 | 1.00 | 0.214 |
|  | ***R*** | - | - | - | - | - | - |
| **TRM** | ***N*** | 24 | 15 | 24 | 27 | 20 | 28 |
|  | ***A*** | 2 | 5 | 2 | 4 | 5 | 7 |
|  | **H_O_** | 0.292 | 0.200 | 0.083 | 0.370 | 0.550 | 0.321 |
|  | **H_E_** | 0.254 | 0.411 | 0.081 | 0.607 | 0.601 | 0.678 |
|  | ***F_IS_*** | -0.150 | 0.523 | -0.022 | 0.395 | 0.087 | 0.530 |
|  | **HWE** | 1.000 | 0.0005 | 1.000 | 0.030 | 0.062 | 0.000 |
|  | ***R*** | - | 0.1415 | - | 0.1414 | - | 0.2067 |
| **DTM** | ***N*** | 16 | 12 | 18 | 17 | 10 | 17 |
|  | ***A*** | 2 | 2 | 2 | 4 | 2 | 6 |
|  | **H_O_** | 0.500 | 0.083 | 0.111 | 0.588 | 0.100 | 0.705 |
|  | **H_E_** | 0.387 | 0.228 | 0.108 | 0.700 | 0.395 | 0.718 |
|  | ***F_IS_*** | -0.304 | 0.645 | -0.030 | 0.164 | 0.757 | 0.018 |
|  | **HWE** | 0.513 | 0.129 | 1.000 | 0.355 | 0.046 | 0.517 |
|  | ***R*** | - | 0.1418 | - | - | 0.2 | - |
